# Nano Zinc Oxide Induced Fetal Mice Growth Restriction, Based on Oxide Stress and Endoplasmic Reticulum Stress

**DOI:** 10.3390/nano10020259

**Published:** 2020-02-02

**Authors:** Bolu Chen, Wuding Hong, Pengfei Yang, Yizhou Tang, Yu Zhao, Zoraida P. Aguilar, Hengyi Xu

**Affiliations:** 1 State Key Laboratory of Food Science and Technology, Nanchang University, Nanchang 330047, China; 407205117055@email.ncu.edu.cn (B.C.); 407205116020@email.ncu.edu.cn (W.H.); hnayypf@126.com (P.Y.); 402313318102@email.ncu.edu.cn (Y.T.); 5603515014@email.ncu.edu.cn (Y.Z.); 2 Zystein, LLC., Fayetteville, AR 72703, USA; zapaguilar@yahoo.com

**Keywords:** zinc oxide nanoparticles, dosage, fetal growth restriction, endoplasmic reticulum stress, reproductive toxicity

## Abstract

ZnO NPs have been assessed to show adverse effects on reproductive organs, but the molecular mechanisms of reproductive toxicity have not been sufficiently studied. In this research, the dosage effects from the oral exposure of ZnO NPs (30 nm) to pregnant mice in gestation day 10.5 to 17.5 was analyzed. Pregnant mice exposed to ZnO NPs induced dam injury, mice fetal growth restriction, and the fetus number decreased. The pathological evaluation showed that ZnO NPs exposure caused placental spongiotrophoblast area decease and structural damage. The RT-qPCR and immunocytochemistry data indicated that ZnO NPs could induce placenta oxide stress, endoplasmic reticulum stress responses, apoptosis, and altered placental function. These findings indicated that ZnO NPs could induce dam injury and fetal growth restriction. Reproductive toxicity of ZnO NPs may be due to placental injury and function alteration caused by apoptosis, oxide stress, and endoplasmic reticulum stress after ZnO NPs exposure.

## 1. Introduction

As a nanomaterial that has been widely used, ZnO NPs (Zinc oxide Nanoparticles) has been used in many areas such as cosmetic, food, paints, etc. [1,2]. Keller et al. reported that ZnO NPs has the highest usage in women’s personal care products compared with all other nanomaterials which led to its release in the environment through domestic wastewater and solid waste after use [3]. These nanoparticles are also added to processed food and to about 3.1% of children’s food [4]. These applications of ZnO NPs raised concerns regarding their effects on reproductive health [5,6,7].

Previous reports claimed that ZnO NPs could induce cytotoxicity. The transcellular (cellular uptake) and paracellular (NanoEL effect) transport of nanomaterials through endothelial barrier have been speculated among the possible ways rendering ZnO NPs toxicity [5,8]. Several recent studies reported that nanomaterials could penetrate the blood-placental barrier [9,10]. Additionally, reports showed that ZnO NPs might induce reproductive toxicity. Hong’s research showed that rat oral administration of 400 mg/kg ZnO NPs caused fetal weight decrease [11]. However, insufficient data could be found regarding the mechanism by which ZnO NPs induce reproductive toxicity.

Previous reports showed that oxide stress and apoptosis were involved in the toxicological molecular mechanism of nanomaterial [12]. What is more, recent research showed that endoplasmic reticulum (ER) stress appeared after nanomaterial exposure which could suggest that ER stress was also in the black box of the molecular mechanism involved in nanomaterial toxicity [13]. Meanwhile, ER stress was also involved in placental development. Du et al. reported that several molecular hallmarks were significantly elevated in placentas of patients with pre-eclampsia [14]. Additionally, studies showed that a high expressional level of the ER stress signal pathway caused reproductive toxicity [15,16]. Therefore, we hypothesized that ZnO NPs would cause reproductive toxicity through the ER stress signal pathway.

In this study, mice were used as in vivo model to study the reproductive toxicity of ZnO NPs. The effects of ZnO NPs were assessed through body weight growth ratio, effects on organs, and hematology. The amount of elemental zinc (Zn) transported to the uterus, placenta, and fetus were also quantified. Fetal development was evaluated by fetal body weight, fetal body and tail length, placental weight and fetal number. The transcription level of key molecular indicators involved in oxide stress, ER stress, apoptosis, and placental function were analyzed by real-time quantitative polymerase chain reaction (RT-qPCR) and verified by immunocytochemistry (IHC). Thus, this research with the intention of providing new sides of understanding the inner mechanism of reproductive was induced by ZnO NPs.

## 2. Methods

### 2.1. Characterization of ZnO NPs

ZnO NPs used in this research were purchased from Xiya Reagent, LLC (Chengdu, China). Particle size was 30 ± 10 nm (information provided by the company). Samples were prepared by dropping the solution of ZnO NPs onto the aluminized paper and the solvent was evaporated. After that, field emission scanning electron microscopy (SEM) with Energy Dispersive Spectrometer (EDS) (JSM 6701F, JEOL Ltd, Peabody, MA, USA) was utilized to characterize the size and morphology of the ZnO particles. Zeta potential was established using the Zetatrac Ultra 151, to determine the average surface charge on the ZnO NPs.

### 2.2. Animals and Treatment

Approximately 8 to 10-week-old female Kunming mice were obtained from the experimental animal center of Nanchang University. The mice were kept at a 12/12 h light/dark cycle condition, libitum supplement food and water at specified times. All processes involving mice used in this experiment adhered to the institutional animal care committee guidelines and this study was approved by the Animal Care Review Committee (approval number 0064257), Nanchang University, Jiangxi, China. At the end of the 1-week adaptation period, female mice were mated with healthy male mice. The day vaginal plug appeared was designated as the gestation day (GD) 0.5. The pregnant mice were randomly divided into five groups (n = 6): Control group, 20, 60, 180, and 540 mg/kg group. The lowest dosage was hundred times as much of dietary reference intakes for zinc recommend by the Chinese nutrition society. The highest dosage was higher than the dosage reported to show an adverse effect to pregnant mice after oral exposure [13]. Mice were exposed to 200 μL/mouse/day from GD 10.5 to 17.5 by oral administration and were observed daily and weighed for clinical manifestations.

### 2.3. Inductively Coupled Plasma Atomic Emission Spectrometry (ICP-AES) Analysis of Zn Content

About 0.5 g of uterus, placenta, and complete fetus were weighed in 100 mL glass beakers, then 10 mL of nitric acid and 2 mL of per chloric acid were added and digest ions were carried out at 240 °C. One hour later, liquid was dried out at 280 °C before allowing the residue to cool down at ambient temperature. Double distilled water was added to the residue to get a final volume of 10 mL. The Zn ion content from each sample was analyzed by ICP-AES.

### 2.4. Histopathological Examination

The placenta were carefully separated from pregnant mice and dipped into 10% formalin solution immediately after harvest. Tissues were sliced into 5–6 μm thickness slices and then mounted on glass slides. These were observed under a Nikon Ti optical microscope (Tokyo, Japan) after hematoxylin-eosin (HE) staining.

### 2.5. Hematological Analysis

The hematological parameters that were evaluated determined the status of pregnant mice in GD 18.5.

### 2.6. Serum sex Hormone Analysis

The serum level of progesterone (Prog) were evaluated. The Prog were evaluated with the Maglumi 2000 Plus automatic analyzer.

### 2.7. RT-qPCR Analysis

AxyPrep Multisource Total RNA miniprep kit (Axygen Scientific, Union City, CA, USA) were used to isolate total RNA from the placenta of the 500 mg/kg group according to the manufacturer’s protocol. cDNA was synthesized with a Takara PrimeScriptTM RT reagent kit (Cat#RR047A, Lot#AK2802) using 1 μg of total RNA following the measured concentration of total RNA using software Quantity One (PDI Inc., New York, NY, USA) which was analyzed with agarose gel electrophoresis. The qPCR primers were synthesized by xiangyin Biotechnology China (Hangzhou China). Quantitative PCR (qPCR) was performed using a SYBR^®^ Premix Ex TaqTM II (TakaRa Code: DRR820A) following the standard protocol with the 7900HT Fast real-time System (Applied Biosystems, Foster city, CA, USA). The thermal cycling program was: One cycle at 95 °C for 1 min, then 40 cycles of 95 °C for 5 s, then 60 °C for 1 min. GAPDH was used as a reference gene, transcription levels were calculated by the 2^−ΔΔCt^ method. Relative gene expression was presented as the fold change, and fold change = 2^−ΔΔCt^.

### 2.8. IHC

The sections of placenta were deparaffinization and rehydration by xylene, 100%/95% ethanol and distilled water. Then, all procedures were performed as Immunohistochemistry Protocol (Paraffin) from the Cell Signaling Technology and incubated overnight at 4 °C with mouse monoclonal antibodies against JNK (Abcam, state abbreviation, USA), then added to biotinylated rabbit anti-mouse IgG secondary antibody. Diaminobenzidine (DAB) as a chromogenic agent showed a positive result. Finally, all slides were imaged under microscopy. Image J 6.0 was used to evaluate the images.

### 2.9. Statistical Analysis

Data were expressed as the mean ± standard deviation (SD) and the one-way analysis of variance (ANOVA) by LSD (SPSS v22.0, SPSS, Inc., Chicago, IL, USA) was used to compare results among different groups.

## 3. Results

### 3.1. NPs Characterization

**Figure 1:**
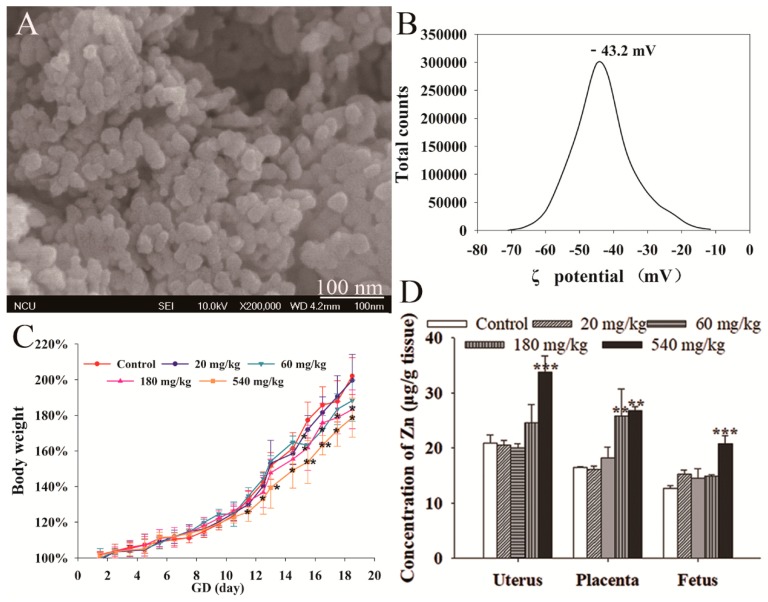
Characterization and maternal effect of Zinc oxide Nanoparticles (ZnO NPs). (**A**) SEM image of ZnO NPs. (**B**) Potential distribution of ZnO NPs. (**C**) Body weight of pregnant mice (n = 6). (**D**) Accumulation of zinc in the organs from GD 18.5 mice (n = 6). These data are presented as the mean and SD * *p* < 0.05, ** *p* < 0.01 vs. control.

The average particle size of ZnO NPs was 30 ± 10 nm. Our previous research reported the size of the ZnO NPs as 27.5 ± 4.1 nm [17]. The SEM micrographs indicated that the shape of nanoparticles was almost spherical (Figure 1A). Additionally, the ZnO NPs exhibited a negative surface charge in water (−43.2 mV) (Figure 1B).

### 3.2. Maternal Effect of ZnO NPs

The maternal effect of ZnO NPs was evaluated through the body weight growth ratio, as well as the hematological and organ coefficient of maternal mice. As shown in Figure 1C, mice exposed to 540 mg/kg ZnO NPs showed significantly lower body weight growth ratio from GD 11.5 (the day after first exposure) to 18.5 compared with the control. Mice in 180 and 60 mg/kg exposure groups showed obvious lower body weight growth from GD 15.5 to 18.5, respectively. The mice exposed to 20 mg/kg ZnO NPs showed the same body weight growth trend as the control group.

As shown in Table S1, the white blood cell (WBC) counts and mean corpuscular hemoglobin concentration (MCHC) in 180 and 540 mg/kg ZnO NPs exposed groups, and platelet counts (PLT) in 540 mg/kg ZnO NPs exposed groups were significantly higher than the control group. Red blood cell distribution width (RDW) in 180 and 540 mg/kg ZnO NPs exposed groups were significantly lower than the control group. In addition, the organ coefficient of thymus showed a decrease in the treatment groups (Figure S1). Meanwhile, no obvious change was found in the Prog content in mice serum after ZnO NPs exposure (Figure S2).

### 3.3. ZnO NPs Distribution

ICP-AES was used to detect the ZnO NPs distribution in the uterus, placenta, and fetus. As shown in Figure 1D, pregnant mice exposed to 540 mg/kg ZnO NPs had significantly higher Zn content in the uterus, placenta, and fetus. The Zn concentration in the placenta in 180 mg/kg ZnO NPs treatment groups was also increased after exposure. There was no difference in Zn content in the placenta, uterus, and fetus among 60 and 20 mg/kg, and control groups.

### 3.4. Fetal Development

**Figure 2:**
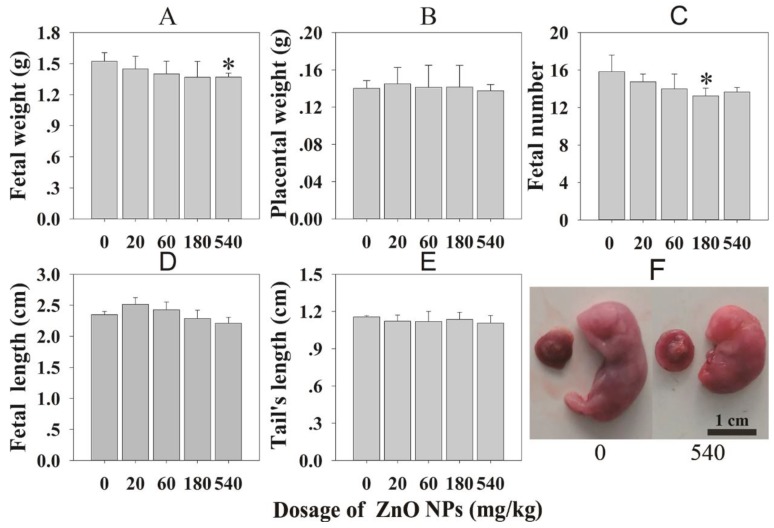
Fetal development status after maternal oral exposure to ZnO NPs. (**A**) Fetal weight; (**B**) placental weight; (**C**) fetal number; (**D**) fetal length; (**E**) tail’s length; (**F**) fetal image. All the data are expressed as the mean ± SD (n = 60). * *p* < 0.05 vs. control.

Fetal development was assessed through changes in the fetal body and tail length, fetal and placental weight, fetal number and malformation rate. Compared with the control group, the weight of the fetuses was significantly decreased in 540 mg/kg treatment groups (Figure 2A), significant reduction in fetal numbers were found in 180 mg/kg treatment groups (Figure 2C). A particular fetus in 540 mg/kg groups showed malformation (Figure 2F).

### 3.5. Placental Histological Analysis

**Figure 3:**
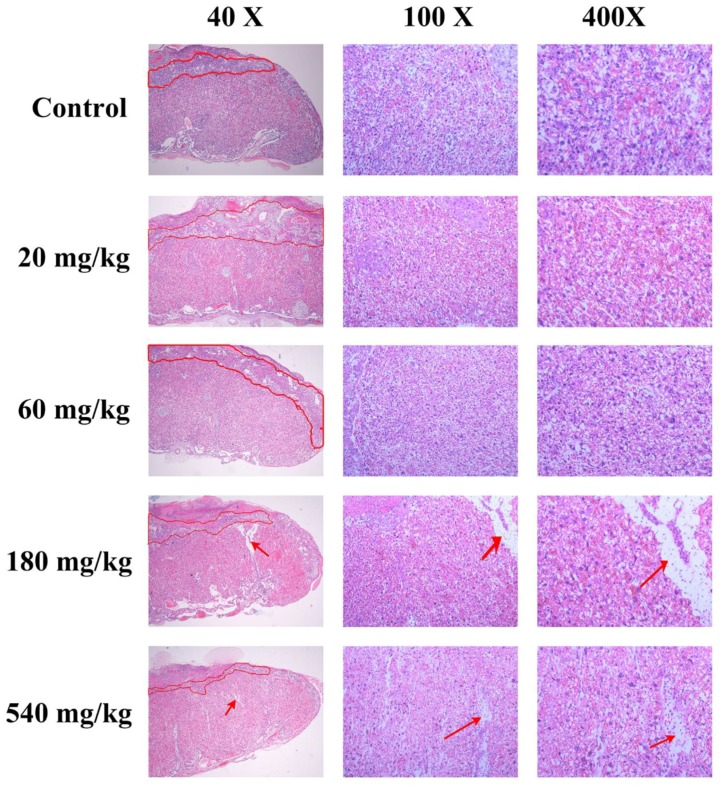
Histological images of the placenta from pregnant mice. The area circled by red circles means spongiotrophoblast, red arrows mean placental structure damage.

The pathological histology of placenta was examined by using HE stains to clarify the effects of dosage of ZnO NPs on fetotoxicity and placental dysfunction. As shown in Figure 3, the placenta of mice treated with 180 and 540 mg/kg ZnO NPs showed variable structural abnormalities. The spongiotrophoblast layer area decreased after ZnO NPs exposure.

### 3.6. RT-qPCR Analysis

**Figure 4:**
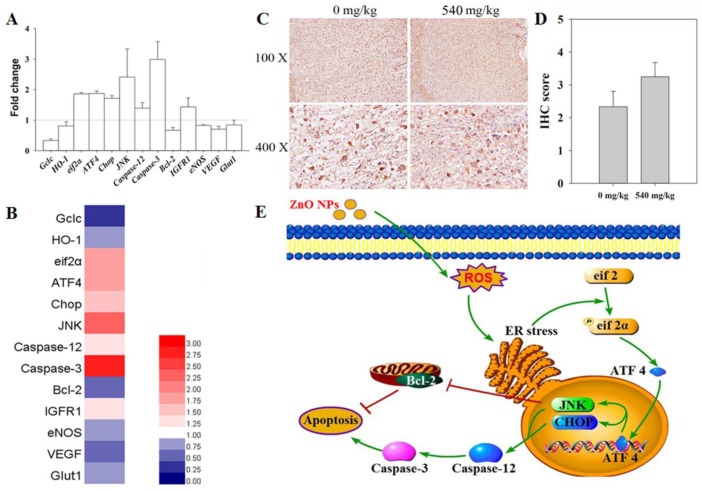
Gene expression after maternal oral exposure to ZnO NPs. (**A**) Relative gene expression of placenta from the 540 mg/kg ZnO groups are presented as the fold change, and fold change = 2^−ΔΔCt^ with GADPH as the reference gene for all test groups (n = 6). (**B**) Hotmap represents a different gene transcription in the placenta. (**C**) Immunohistochemistry (IHC) results show Jun N-terminal kinases (JNK) expression in placenta among the control group and 540 mg/kg groups. (**D**) The IHC score of JNK. (E) Schematic drawing of the proposed mechanism for ZnO NPs that induce placenta injury.

In the control and 540 mg/kg treatment groups, the transcription level of genes related to oxide stress, ER stress, apoptosis, hormonogenesis, growth factors, and glucose transport were tested in the placenta to investigate the mechanism involved in ZnO NPs-induced maternal and fetal development (Figure 4A). Genes related to oxide stress (glutamate-cysteine ligase catalytic subunit (Gclc), heme oxygenase 1 (HO-1) showed downregulation. Meanwhile, the genes related to ER stress (eukaryotic initiation factor 2α (*eif2α*), activating transcription factor 4 (*ATF4*), C/EBP homologous protein (*Chop*), c-Jun N-terminal kinases (*JNK)*), and apoptosis-promotion (*Caspase-12*, *Caspase-3*) showed transcription upregulation. The genes related to anti-apoptosis (B-cell lymphoma-2 (*Bcl-2*)), growth factor (Endothelial NOS (*eNOS*), vascular endothelial growth factor (*VEGF*)), and glucose transport (*Glut1*) showed transcription downregulation. The insulin-like growth factor 1 receptor (*IGFR1*) showed transcription upregulation. The Hotmap showed the changes in the gene profile (Figure 4B).

### 3.7. IHC

To confirm the expression level of proteins belonging to the ER stress signal pathway in the placenta, IHC was used. As shown in Figure 4C,D, the expression of JNK was upregulated in the 540 mg/kg ZnO NPs treatment groups.

## 4. Discussion

ZnO NPs have been widely used in many areas, especially in female care products [3]. However, with the widening use, the risk of exposure to ZnO NPs could also increase. During the pregnancy stage, harmful effects of new products such as those containing ZnO NPs, affect the reproductive route inducing fetal malformation or even fetal death. Recently, some researches showed that ZnO NPs could cause reproductive and development toxicity, but insufficient studies had been devoted to determination of the internal mechanism involved in the reproductive toxicity of ZNOs [7,18,19].

Body weight, hematology, and organ weights are the most common and most sensitive indicators of the harmful effects of drugs on animals. These were adopted in this study to show the relative ZnO NPs dose-exposure effects. The body weight of pregnant mice showed significant reduction in the growth rate of those exposed to 180 and 540 mg/kg ZnO NPs while none were observed among those exposed to the lower dosages at 20 and 60 mg/kg. These observations were in line with previous results reported by Hong et al. [11]. In addition, the hematological results showed a significant increase in WBC and PLT counts in those exposed to higher doses of ZnO. These observations were in accordance with previous researches which indicted that exposure to NPs could induce inflammation in mice [20,21]. The organ coefficient data showed a significant decrease in thymus weight/body weight which implied that the exposure to ZnO NPs may have disturbed the inflammation system during pregnancy that was consistent with the hematological results. While no obvious change of Prog was found, which may indicate that the reproductive toxicology of ZnO NPs may not contribute to hormone expression.

The distribution of ZnO NPs in the reproductive system was also investigated. Pregnant mice exposed to 540 mg/kg ZnO NPs could cause the zinc level to increase in the uterus, placenta, and fetus. The biodistribution of ZnO NPs in the reproductive organs may indicate that the nanoparticle could be transported to other tissues and organs after uptake by the gastrointestinal tract [22].

In order to have normal embryonic growth, it is necessary to have a complete placenta structure and normal function. Previous researches showed that placental dysfunction could cause fetal growth restriction and miscarriage [23]. Mature murine placenta was considered to consist of Labyrinth, spongiotrophoblast, trophoblast giant cell, and maternal decidua. In this research, a high dose of NPs exposure showed placental structure damage and decrease in spongiotrophoblast layer. This indicated that exposure to the NPs may have damaged the normal structure of the placenta which may have affected the placental function.

Some indexes were chosen to determine the dose related fetotoxicity of NPs. Studies have shown that exposure to high doses of NPs reduced the fetal number and decreased fetal weight which were according to the previous study. Teng et al. discovered that the organogenesis period (GD 7–16) was more vulnerable to such toxicity compared with the peri-implantation period (GD 1–10) of pregnancy. At the same time, they found that ZnO NPs could affect placental weight, fetal rat weight, and vitality in the 200 mg/kg group [24].

To find out the mechanism by which ZnO NPs induce reproductive toxicity, the changes in representative gene expression in the placenta were evaluated in the control group and the 540 mg/kg group on GD 18.5. Some key gene relative to oxide stress (*HO-1*, *Gclc*), ER stress (*eif2α*, *ATF4*, *Chop*, *JNK*), apoptosis (*Caspase-12*, *Caspase-3*, *Bcl-2*), growth factor (*IGFR1*, *eNOS*, *VEGF*), and GLU transport (*Glut1*) were chosen. HO-1 and Gclc is the gene that had been reported as the indicator of oxide stress induced by nanomaterials [25,26]. The downregulation in these two genes could be indicative that ZnO NPs may induce oxide stress which could induce ER stress during which eif2 would be phosphorylated. When eif2α is transformed into phosphorylated eif2, it could inhibit the combination of eif2 and base, which could in turn inhibit the translation process [27]. When eif2 is being inhibited the ribosome could jump over the open reading frame 1 (ORF1) and combine with the ORF2 area of ATF4 which could begin the translation process of ATF4 [28]. ATF4 is a transcription factor which could activate JNK and CHOP transcription [28]. The IHC results indicated that JNK was transcribed and expressed after 540 mg/kg ZnO NPs exposure. These two genes had been reported to induce apoptosis [29]. Meanwhile, the genes which exhibited an increase could be indicative of the ZnO NPs induction of placenta ER stress. Caspase 12 is activated by ER stress which activate the Caspase 3 which begins the process of apoptosis. Bcl-2 is a protein which is anti-apoptosis. The results showed that *Caspase 12* and *Caspase 3* transcription were upregulated and *Bcl-2* was downregulated. These results indicated that ZnO NPs may have induced ER stress which lead to cell apoptosis. To further explore if the placental function was disturbed by the ZnO NPs exposure, transcription level changes of genes related to growth factors and glucose transport were examined. The growth factor and glucose transport gene showed downregulation which could indicate that the placental function was disturbed. Meanwhile, the IGFR1 showed upregulation which could have indicated that the placental structure was damaged and rebuilt which also require additional studies.

Thus, our preliminary results showed that ZnO NPs initiated the oxide stress–ER stress events and enhanced apoptosis and placenta function injury which eventually led to reproductive toxicity (Figure 4D). However, most of our molecular results were based on transcription levels of several genes, therefore, a more systematic research into more genes and protein expression are needed to verify our observations and to reach a better understanding of the molecular mechanism of the reproductive toxicity induced by ZnO NPs.

## 5. Conclusions

In this study, we focused on the reproductive toxicity of oral exposure to different dosages of ZnO NPs in pregnant mice. Our finding suggested that oral exposure to more than 180 mg/kg ZnO NPs during GD 10.5 to 17.5 could induce maternal injury, fetal growth restriction, and fetal number decrease. Meanwhile, treatment with 540 mg/kg could induce elemental zinc to cross the blood placental barrier. ZnO NPs could induce reproductive toxicity through the oxide stress and ER stress that resulted in induction of placental apoptosis and placental function injury. To have a better understanding of the molecular mechanism of reproductive toxicity induced by ZnO NPs, the study of protein expression levels must be accompanied with transcriptomics, proteomics, and metabolomics.

## 6. Patents

This section is not mandatory, but may be added if there are patents resulting from the work reported in this manuscript.

## Supplementary Materials

The following are available online at https://www.mdpi.com/2079-4991/10/2/259/s1. Figure S1: Organ coefficient from female mice treated with various dosages of ZnO nanoparticles and control. All the data are expressed as the mean ± SD. * *p* < 0.05, ** *p* < 0.01 vs. control (n = 6); Figure S1: Hormone content in serum. (A) FSH, (B) Prog. All the data are expressed as the mean ± SD. * *p* < 0.05, ** *p* < 0.01 vs. control (n = 3); Table S1: Whole blood analysis from female mice treated with various dosages of ZnO nanoparticles and control.
