# Supplementary material for: Nano Zinc Oxide Induced Fetal Mice Growth Restriction, Based on Oxide Stress and Endoplasmic Reticulum Stress
Source: Nanomaterials (Basel). 2020 Feb 2;10(2):259. doi: 10.3390/nano10020259 (PMC7075166; doi:10.3390/nano10020259)
Supplement: Supplementary file 1 [file nanomaterials-10-00259-s001.pdf]

Supplemental Materials

Table S1. Whole blood analysis from female mice treated with various dosage of ZnO nanoparticle and control.

	0	20 mg/kg	60 mg/kg	180 mg/kg	540 mg/kg
RBC × 10^{12}/L	7.73 ± 0.44	7.82 ± 0.2	8.04 ± 0.78	7.22 ± 1.08	7.97 ± 1.27
HGB	113.75 ± 9.28	118.5 ± 3.91	122.33 ± 16.22	109.33 ± 19.55	121.67 ± 18.52
HCT	41.03 ± 3.55	41.13 ± 1.18	43.43 ± 6.88	36.7 ± 6.38	39.93 ± 5.97
MCV	52.98 ± 2.37	52.65 ± 2.27	53.87 ± 3.81	50.67 ± 2.29	50.27 ± 1.75
MCH	14.68 ± 0.58	15.18 ± 0.43	15.2 ± 0.62	15.07 ± 0.62	15.3 ± 0.28
MCHC g/L	277.5 ± 3.57	288 ± 8.92	282.33 ± 9.34	297.67 ± 3.2 **	304.67 ± 5.19 ***
RDW	19.93 ± 0.6	19.03 ± 0.68	19.17 ± 1.19	18.33 ± 0.84 *	17.63 ± 0.17 **
WBC × 10^9/L	5.68 ± 2.31	4.2 ± 1.07	9.4 ± 3.85	8.37 ± 1.16 **	7.23 ± 1.96 *
PLT × 10^{12}/L	850 ± 235.75	755.25 ± 154.59	686.33 ± 204.93	755.67 ± 234.27	1122.33 ± 126.15 **

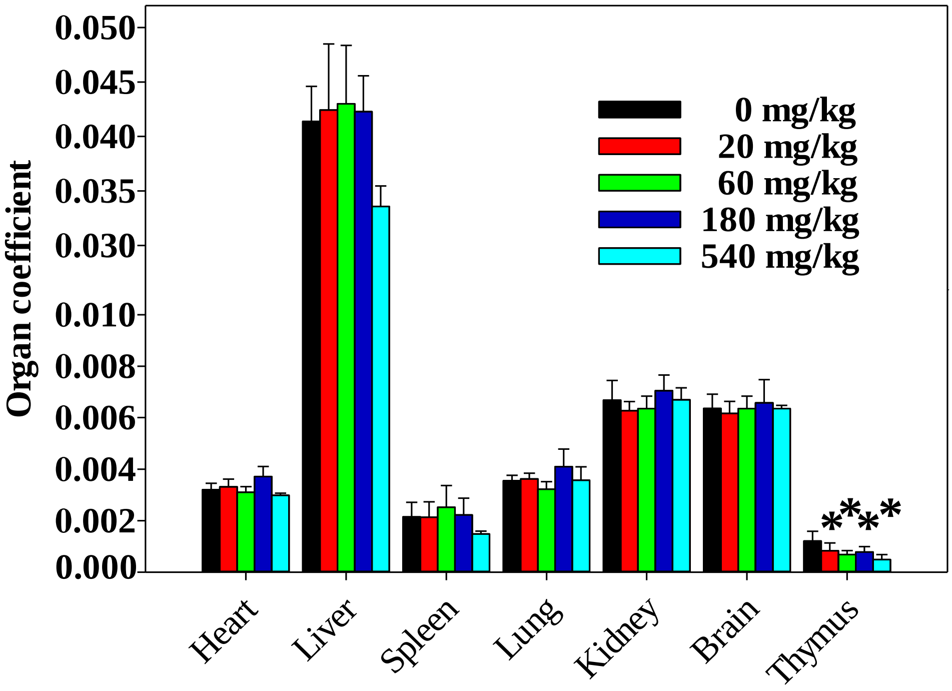

Figure S1. Organ coefficient from female mice treated with various dosage of ZnO nanoparticle and control. All the data are expression are express as mean ± SD. *P < 0.05, ** P < 0.01vs. Control. (n = 6).

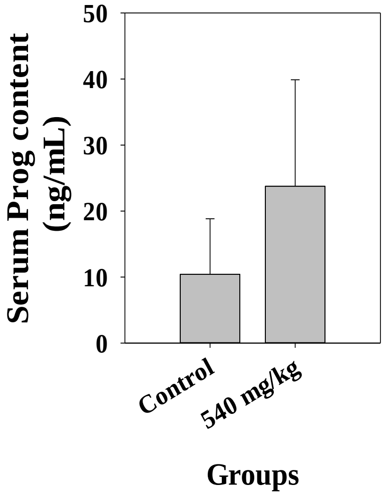

Figure S2. Prog content in serum. All the data are expressed as mean ± SD (n = 3).
